# Entomopathogenic nematode-gastropod interactions

**DOI:** 10.21307/jofnem-2021-061

**Published:** 2021-06-29

**Authors:** Jacob Schurkman, Adler R. Dillman

**Affiliations:** 1 Department of Nematology, University of California, Riverside, 900 University Ave, Riverside, CA, 92521

**Keywords:** Biological control, Entomopathogenic nematode, Gastropod, Host-parasite interaction, Management, Methodology

## Abstract

Entomopathogenic nematodes (EPNs) infect and kill insects and have been successfully used in the biological control of some insect pests. Slugs and snails are known to be significant pests of agriculture and serve as vectors for disease-causing microbes that can affect crops and humans. The potential of EPNs to be used in the biological control of gastropods has not been well-studied. The few studies that have been performed on the efficacy of EPNs in controlling gastropod pests and vectors were reviewed. Suggested criteria for further assessments of EPN-gastropod interactions are: Dose of EPNs used, length of infection assays, host biology, nematode biology and development, and Koch’s postulates. There are provocative data suggesting that EPNs may be useful biological control agents against gastropod pests of agriculture and vectors of disease, though additional studies using the suggested criteria are needed, including the publication of negative data or studies where EPNs were not efficacious or successful in controlling gastropods.

Parasitic nematodes infect a variety of invertebrates and are used in biological control. Entomopathogenic nematodes (EPNs), a guild of insect-parasitic nematodes, have been used with some success in the biological control of insects. These nematodes have been shown to be pathogenic to insects and are considered to have few non-target effects, although it must be noted that specificity studies and non-target infection experiments are few (Bathon, 1996; Piedra-Buena et al., 2015; Sandhi and Reddy, 2019). EPNs are associated with insect-pathogenic bacterial symbionts. The bacterial species in question do not occur in nature without a nematode associate, and the exact identity of each bacterial symbiont is dependent on the species of their particular nematode partner. *Photorhabdus asymbiotica* is an exception, as it occurs in both free-living and symbiotic conditions, and can be pathogenic to mammals, including humans. For example, all species of the nematode genus *Steinernema* associate with *Xenorhabdus* bacterial species, and all species of the nematode genus *Heterorhabditis* associate with *Photorhabdus* bacterial species (Kaya and Gaugler, 1993; Lewis et al., 2006). While most studies focus on these primary associations, some studies suggest that EPNs are associated with several secondary bacterial strains, although the significance or potential mutualistic nature of these additional associations is not well understood (Babic et al., 2000; Enright and Griffin, 2004; Kim et al., 2009; Ogier et al., 2020).

As with many skin-penetrating nematodes that infect mammals, EPNs are only infectious to insects when they are in the infective juvenile (IJ) life stage, an alternate L3 stage similar in many anatomical respects to the dauer juvenile in *C. elegans*. EPN IJs emerge from a resource-depleted insect cadaver to seek a new host using various behavioral strategies. If successful in making contact with a susceptible host, they enter its hemocoel and then release their symbiotic bacteria, along with a cocktail of nematode proteins that are known or assumed to implement a variety of infection-facilitating processes (Chang et al., 2019; Lu et al., 2017). This multipronged assault typically results in rapid host death, usually within five days (Dillman et al., 2012). The IJs then feed on the multiplying bacterial symbionts and the cadaver’s decomposing body contents, mature into adults, and several rounds of nematode reproduction ensue. When the cadaver’s nutritional resources begin to reach depletion, the youngest cohort of developing nematodes become IJs and emerge from the host to repeat the life cycle again (Dillman et al., 2012; Kaya and Gaugler, 1993). The IJs are developmentally arrested and only resume feeding and development after they have infected a new host. This process of resumption is linked with the initiation of the parasitic phase of their life cycle, these processes are jointly referred to as activation (Alonso et al., 2018; Lu et al., 2017).

Insects are not the only agricultural and health pest that can be targeted by biological control agents. Some important diseases as well as agricultural damage are caused by gastropods. Terrestrial gastropods serve as hosts and vectors for pathogens like *Alternaria brassicicola*, members of the family *Peronosporaceae*, and other plant pathogenic fungi (Hasan and Vago, 1966; Turchetti and Chelazzi, 1984; Wester et al., 1964). Some species of gastropods can harbor human pathogens as well. Slugs and snails may have been partially responsible for widespread recalls of spinach and other salad crops as suggested by the discovery of *Campylobacter* spp. and *Escherichia coli* on sampled gastropods (Raloff, 2007; Sproston et al., 2006). Multiple terrestrial gastropods have also been found to carry *Angiostrongylus cantonensis*, a rodent-parasitic nematode that is also known as the rat lung worm which causes eosinophilic meningitis in humans (Iwanowicz et al., 2015; Lindo et al., 2004; Teem et al., 2013). The possibility that nematodes could be used to control gastropods has been largely unexplored. Most research on nematode agents against gastropods has focused on *Phasmarhabditis* nematodes, which are known to infect snails and slugs (Ross et al., 2012; Wilson et al., 1993). This review explores the meager scientific literature relating to EPNs and their potential parasitic relationship to gastropods, including possible functional parallels with gastropod-parasitic nematodes not known to infect insects.

## Nematode interactions with insects and gastropods

Nematodes associate with insects and gastropods in a variety of ways. These interactions include phoresy, necromeny, and parasitism, among others (Sudhaus, 2008). Animal-parasitic nematodes almost invariably evolved from free-living ancestors, which fed on bacteria and other microbes (Blaxter and Koutsovoulos, 2015). Many of these free-living nematodes rely on ephemeral environments such as rotting fruits or decomposing plant material. One way in which they facilitate movement between environments is phoresy, where the nematodes use other organisms for transport (Sudhaus, 2008). Phoretic associations are common among nematodes and invertebrates. *Caenorhabditis elegans*, a free-living nematode, has been reported to travel from mushroom to mushroom on flies, and has phoretic associations with many other invertebrates, including snails, slugs, isopods, and chilopods (Petersen et al., 2015; Rinker and Bloom, 1982).

It is hypothesized that some of the nematode lineages that utilized phoresy subsequently evolved adaptations to utilize their phoretic host’s resources through a more unusual form of mutualism known as necromeny. In a necromenic association, the nematodes do not actively contribute to the death of the host, but they wait inactively inside the host or on its body surface until it dies. Upon death they become active to utilize the resource-rich cadaver itself, while also feeding on invading bacteria and yeasts (Sudhaus, 2008).

Parasitism is a relationship in which one organism has a metabolic association with another, but of a clearly antagonistic rather than mutualistic nature: the first benefits from the relationship at the expense of the other, by feeding on the host’s tissues and nutrients while the latter remains alive (Loker and Hofkin, 2015). In nematodes of the infraorders Rhabditomorpha and Panagrolaimomorpha, dauer juveniles and phoretic adaptations are common in many species. In these two groups, parasitism of animals is thought to have evolved repeatedly from necromeny in arthropods and/or other invertebrate hosts, beginning with facultative parasitism (opportunistically parasitic), and then eventually leading to obligate parasitism (strict parasites) (Dillman et al., 2012; Sudhaus, 2008). Described species of EPNs are obligate parasites that require live insects hosts which they then must kill to complete their life cycle inside the cadaver. Studies done on the effects of EPNs on non-target organisms have found that some EPNs can infect or otherwise affect non-target invertebrates and vertebrates such as slugs, snails, earthworms, and frogs that are beneficial or neutral to agricultural environments (Capinera et al., 1982; Li et al., 1986; Poinar and Thomas, 1988). When the potential impacts of EPNs on non-targets were assessed by Akhurst and Smith (2002), they found that EPNs could have indirect and/or direct effects on beneficial predators and parasitoids, or other invertebrate organisms (Akhurst and Smith, 2002). Some EPNs could kill non-arthropods (direct), or they could parasitize the hosts of the predators and parasitoids which would reduce their food source (indirect). However, additional research into EPN interactions with potential or known non-target hosts is needed.

## Criteria for evaluating nematode-gastropod associations

There are some important aspects that should be considered in assessing the potential of EPNs or other nematodes in the biological control of gastropods. This will ensure that the nature of the interaction between nematode and gastropod (phoretic, necromenic, etc.) can be determined and the full potential of the nematodes in biological control can be evaluated.

1. The dose of the nematodes used for virulence assays is critical and should be calculated. The dose should be economical to determine whether the EPNs could be used advantageously in the field. The recommended dosage for EPN application in the soil is 25 IJs/cm^2^ (Piedra-Buena et al., 2015). However, the dosage recommended by manufacturers varies. For example, Arbico Organics recommends that 5 million IJ’s should be used per 150 m^2^. This is approximately 3 IJs/cm^2^. This dose would cost about $61.00 (USD), per 150 m^2^, according to the company website.
2. The timing of the assay should be appropriate for the culture medium and arena being used. An assay done in a lab should not continue for months to measure mortality. Instead, a lab assay should only extend for an economically reasonable period of time in order to establish whether the EPN can be used as a biological control agent and is able to kill pests in a reasonable time period. A field trial might more reasonably be conducted over a longer period of time to determine not only the short-term consequences but also the long-term viability of the nematodes under local conditions of soil properties, climate, other crop/soil management techniques applied by growers, etc.
3. Aspects of host biology should be measured. These aspects include mortality, reproduction, weight, size, and other features, ideally including assessments of immunity versus susceptibility, reproductive changes or other measures of health. We suggest that it is necessary to determine whether the nematodes are able to kill the host, whether hosts exposed to the nematodes are able to reproduce, and if so, then how much or how little reproductive deficit the exposed host population incurs compared to untreated host populations. If the target pest populations are able to survive and reproduce in the presence of the control agent, then the value of the candidate control agent is most likely diminished (barring the possibility of additional factors in the field that cannot easily be replicated in laboratory assays, such as synergies with other control methods because they are known to cause harm to protected flora or fauna, etc.).
4. Aspects of nematode biology should be measured. These aspects include whether the nematodes are activated by exposure to the host, whether the nematodes develop or mature over the course of infection, and whether the nematodes reproduce in or outside of the host. If the applied nematodes do not mature inside of the host, this may suggest that the nematodes have a phoretic or necromenic relationship with the host, rather than a parasitic one (Dillman et al., 2012; Sudhaus, 2008). If the nematodes are not activated while on or inside the host, this may also suggest a non-parasitic relationship.
5. Koch’s postulates remain effective for evaluating parasitic nematodes and should be used (Berman, 2019). The gastropods should be inoculated with nematodes, and the nematodes should be retrieved and isolated from the gastropods post infection. Gastropods which were inoculated with nematodes should have negatively altered biology, while control gastropods should remain healthy. Nematodes should then be isolated from dead or severely morbid hosts at the end of the first course of infection, and be used to repeat the infection protocol in order to confirm that the same course of pathology develops in another trial group of naïve hosts. The fulfillment of Koch’s postulates may not be necessary to determine whether a nematode can be used as an efficient biological control agent. It is possible for a nematode to invade a gastropod, release pathogenic bacteria, and kill the gastropod. After gastropod death, the nematode may be unrecoverable due to an unstable environment, therefore leaving Koch’s postulates unfulfilled. However, the nematode could still be an effective biological control agent due to causative pathogenicity. The fulfillment of Koch’s postulates is necessary however when determining nematode host-parasite interactions and establishing the nematode as a bona fide parasite.

## Studies of EPN-gastropod interactions

The first study to evaluate the interaction between EPNs and gastropods was published by Li et al. (1986) (Table 1), focusing on the snail *Oncomelania hupensis*, an intermediate host of *Schistosoma japonicum* (blood flukes). Because this paper was not available in English but is cited by most subsequent studies in this field, we summarized this report in a higher level of detail than the other papers reviewed here. Li et al. first attempted to infect *O. hupensis* with five different species of EPNs: *Steinernema glaseri, S. feltiae, S. bibionis* (junior synonym of *S. feltiae*)*, Heterorhabditis heliothidis* (junior synonym of *H. bacteriophora*) and *H. heliothidis* T327 (junior synonym of *H. bacteriophora*). Only 10 snails were tested per treatment and the experiments were not replicated. They found that *S. glaseri* killed nine snails and recovered nematodes from all nine cadavers, while *S. feltiae* killed five and all five cadavers contained nematodes, *S. bibionis* killed six snails and three cadavers contained nematodes, *H. heliothidis* killed five snails but none of the cadavers contained nematodes, *H. heliothidis* T327 killed nine snails but only cadaver one contained nematodes, and a water control killed no snails.

The authors then focused on *S. glaseri* and performed a series of virulence assays where they tested the mortality of *O. hupensis* snails exposed to *S. glaseri*. They reported that at 340 IJs/cm^2^ the mortality of snails was between 70 and 97%, with ~97% of dead snails containing *S. glaseri*. Then they tested different concentrations of *S. glaseri* IJs (50, 100, 150, 200, and 300 IJs/cm^2^) in flowerpots filled with wetted soil and populated by 50 snails. This is equivalent to 314 IJs, 628 IJs, 942 IJs, 1,256 IJs, or 1,884 IJs per snail, respectively. The recommended dosage for insect control with EPNs is typically about 25 IJs/cm^2^ ((Piedra-Buena et al., 2015). High doses (200 and 300 IJs/cm^2^) resulted in a mortality rate above 90%. The dosage of 150 IJs/cm^2^ had a mortality rate of 83.5%. Doses of 100 and 50 IJs/cm^2^ resulted in 47 and 45% mortality, respectively. Doses less than 50 IJs/cm^2^, which would be nearer to the recommended dose, were not tested.

The work of Li et al. (1986) provided the first evidence that EPNs may be suitable biocontrol agents against gastropods, and reported that *S. glaseri* is the most capable of successfully killing *O. hupensis* of the EPNs tested. It also demonstrated the economic feasibility of using EPNs to control disease vectors. To treat the area in the pots used in the study (314 cm^2^) with EPNs from Arbico Organics at a dose of 200 IJs/cm^2^, which provided above 90% mortality, the effective cost would be approximately $0.76 per pot, based on current pricing. This could be a financially viable option for high-value plant production, for instance of premium perennial ornamentals such as orchids. However, the study does not prove that *S. glaseri* has a parasitic relationship with *O. hupensis*. It also does not support the conclusion that EPNs in general are capable of infecting *O. hupensis*. They reported finding nematodes in the *O. hupensis* cadavers. However, they did not stage the nematodes, leaving open the possibility that the nematodes could still have been IJs from the initial inoculation. The nematodes may not have developed or reproduced inside of the hosts. If this were the case, *O. hupensis* may be considered a paratenic host for *S. glaseri* because the nematodes were not able to complete all or part of their life cycle inside or on the host. Li et al. (1986) did not measure any aspect of host biology other than survival. They could have measured host traits such as the weight, size, gender, or age of the snails. It is possible that *S. glaseri* was only able to successfully kill younger snails. This type of effect is seen with *Phasmarhabditis hermaphrodita*, which is only able to effectively kill juvenile *Helix aspersa* and is not capable of killing adults ((Williams and Rae, 2015).

The next study which examined possible antagonisms between gastropods and EPNs was done by Jaworska (1993). This brief note claimed that *S. carpocapsae*, *S. feltiae,* and *H. bacteriophora* were capable of infecting, killing, and developing in the gray field slug *Deroceras reticulatum* (Jaworska, 1993), but the experiments were not replicated, and the methodology was described too briefly to be meaningfully considered here further.

A study by Wilson et al. (1994) sought to replicate the results of Li et al. (1986) with a few different species of EPNs. In this study the researchers also compared the efficiency of EPNs at killing the highly pestiferous *D. reticulatum* with that of the gastropod-parasitic nematode *Phasmarhabditis hermaphrodita*. They performed virulence assays using *P. hermaphrodita*, *Heterorhabditis sp.*, and *S. feltiae* against *D. reticulatum* in 9 cm petri dishes with filter paper, following the standard EPN assay protocol as used with *Galleria mellonella* larvae. They also performed virulence assays in soil. It is not clear why *S. feltiae* was used instead of *S. glaseri* since previous work found that *S. glaseri* was able to efficiently kill *O. hupensis*. This study also tested the nematodes’ virulence against the larvae of the darkling beetle *Zophobus morio*. EPNs have largely been reported as insect-specific parasites, and as safe for non-target hosts, although additional research into their interactions with potential or known non-target hosts is needed (Georgis et al., 1991; Kaya and Gaugler, 1993; Sandhi and Reddy, 2019). The inclusion of *Z. morio* as a host in this study served to determine whether the tested EPNs are more efficient at killing insects than gastropods. In addition to these virulence assays, they tested whether the bacterial associates of the EPNs in the study, *Photorhabdus luminescens, Xenorhabdus luminescens*, and *Xenorhabdus bovienii*, could kill *D. reticulatum* when injected. These bacterial species are highly pathogenic to susceptible insects and contribute to the death of insect hosts in EPN infections. The nematodes also contribute via their excreted/secreted proteins (Chang et al., 2019; Kenney et al., 2019; Lu et al., 2017).

In the petri dish virulence assays, Wilson et al. (1994) used approximately 50 IJs/cm^2^ along with 10 *D. reticulatum* slugs per plate. The assays were performed at both 14 and 23°C. There was significant mortality of control slugs in all conditions, but it was worst in petri dishes at 23°C, where they reported 50% death of control slugs after 6 days. Nonetheless, *P. hermaphrodita* was significantly more effective at killing slugs than the EPNs in petri dishes at 14°C, and in soil. For the insect bioassay, the EPNs were significantly more effective at killing *Z. morio* than *P. hermaphrodita*, killing >60% of the insects whereas *P. hermaphrodita* killed <5%. Their findings indicated that the EPNs are far better suited as biocontrol agents against insects like *Z. morio*. In the bacterial injection experiments, they reported low pathogenicity of the EPN-associated bacteria and concluded that neither *Heterorhabditis sp.* nor *S. feltiae* had potential as biological control agents against *D. reticulatum*. After this report, enthusiasm waned and little if any research followed on EPN-gastropod associations for the next 20 years.

Investigations of EPN-gastropod associations resumed in 2014 however, with a report on physiological alterations in the snail *Bradybaena similaris* induced by the EPN *Heterorhabditis indica* LPP1 (Tunholi et al., 2014). *B. similaris* is a serious agricultural pest and the research report investigated whether *H. indica* could negatively affect the snail. This report is the first to use *H. indica* to investigate EPN-gastropod associations. It is unclear why this particular nematode was used instead of something that had been tested in previous studies. The infection step of the experiments in this paper was unusual and not quantified by adding a known amount of EPN IJs to the soil in the infection arenas. Instead, they added to each arena 6 *G. mellonella* cadavers infected with *H. indica*, and then 16 snails. *H. indica-*infected waxworm cadavers can yield 191,922 (+34,192) IJs each (Pradeep, 2016), meaning that the 16 snails per arena were probably exposed to over 1.1 million IJs in a small arena, which is nearly 200,000 IJs per snail (Pradeep, 2016). A dose this high confounds any meaningful conclusions for field applications, where feasible EPN applications require their formulation as liquid suspensions or in diatomaceous earth pellets, after high-yield nematode cultivation in fermenters rather than in waxmoth larvae. Additionally, this study provided no evidence that the snails were infected by EPNs, but rather that the snail biology is affected when they are exposed to extreme quantities of EPNs. Previous work has shown that *Biomphalaria* snails respond to the mere presence of schistosome miracidia by accelerating their egg production as a fecundity compensation mechanism (Minchella and Loverde, 1981), regardless of whether or not they are actually infected. Hence, dissection of exposed hosts is needed to confirm infection, as changes in host biology alone are not necessarily sufficient to demonstrate infection. In future studies it will be important to evaluate activation and development of the IJs. EPN IJs are non-feeding and the mouth is sealed (Ciche, 2007). IJ activation is the process of resuming development from the dauer-like state of arrested development (Hawdon et al., 1995; Lu et al., 2017; Toubarro et al., 2009). After activation, the resumption of development and reproduction in a potential host would be stronger evidence of a parasitic association between nematode and host if a reasonable dosage is used.

Another study on *H. indica* interactions with *B. similaris* was published by Tunholi et al. (2017a). Unfortunately, the same method was used, where each snail was exposed to ~200,000 IJs, confounding any likely relevance to the much lower nematode dosage levels required for cost-effective biological control, especially so at the large scales of acreage needed for commercially viable production of produce crops and ornamentals. Application aspects aside, this study used physiological assays to assess host biology by obtaining the first post-exposure measurements of glucose, glycogen, galactogen, pyruvic acid, and lactic acid concentrations in exposed snail tissues, as well as lactate dehydrogenase activity (LDH) and the post-exposure fecundity of *B. similaris* (Tunholi et al., 2017a). Including biochemical measures of snail health was an important contribution of this paper and lays a foundation for future studies.

There was a second study in 2017 assessing the use of the EPN *Heterorhabditis baujardi* to control the snail *Lymnaea columella* (Tunholi et al., 2017b). In this paper, a more appropriate method of exposing the snails to EPNs was used, with 150 IJs and a single snail in a 24-well plate, though this is still higher than the recommended dose. The aim of the study was to assess whether *H. baujardi* LPP7 could be utilized as an alternative for biological control of fascioliasis, a disease caused by flukes which infect *L. columella* as an intermediate host.

After exposure to EPNs, snails were placed in aquariums and fed lettuce leaves every other day. Mortality was then recorded for 3 weeks. Host reproduction and host galactogen was measured, and histology was performed on the snails to assess the putative infections. The mortality rate of the snails exposed to *H. baujardi* was low by day 7 post infection (~15%), but eventually reached 66.6% after 3 weeks, which is about 10% higher than what was recorded in experiments with *H. indica* (Tunholi et al., 2014, 2017a). A hallmark characteristic that distinguishes EPNs from other insect-parasitic nematodes is that they kill insect hosts within 5 days post infection (Dillman et al., 2012). The cause of the lag in mortality in this study was not determined and no speculation was made. Exposure to *H. baujardi* led to decreased egg masses laid during the 3-week period. Exposed snails also had significantly lower concentrations of galactogen present in the albumen gland, indicating metabolic stress from less energy reserves. The histological analyses revealed no alterations to the gonadal tissues of the snails, but did show the presence of intense cell disorganization, with granulomas in multiple tissues like the cephalopodal mass and the digestive gland where larval stage EPNs were present. However, the life stage and condition of the EPNs found was not reported.

The question of whether *H. baujardi* is capable of being used as an efficient biological control of *L. columella* remains open, though this report does provide evidence that EPN-exposed snails are negatively affected by this contact. However, for *H. baujardi* to work as an efficient biological control agent, it must be capable of killing *L. columella* in their common inhabited environments, preferably in a shorter time frame than 3 weeks after exposure to the nematodes. This task may prove difficult as the snails are largely aquatic, and do not frequently reside above the waterline, while *H. indica* primarily lives in terrestrial conditions. Thus, additional experimentation is needed before any conclusions regarding the value of EPNs in controlling *L. columella* or other gastropod vectors of disease can be made.

The most recent study of EPN-gastropod interactions was by Saeedizadeh and Niasti (2020). This paper explored the response of *Parmacella ibera* to the EPNs *S. feltiae*, *S. carpocapsae* and *H. bacteriophora*. *P. hermaphrodita* and metaldehyde bait were used as controls. Metaldehyde baits are molluscicides which are applied to the soil to dehydrate gastropods upon consumption. These baits are non-targeted, and are toxic to mammals, and other organisms, including those that may be beneficial to crops (Dolder, 2003). These types of molluscicides are commonly available and widely used. Feeding activity and mortality of exposed slugs were measured along with damage or grazing incidence of the slugs when exposed to the nematode treatments and molluscicide bait.

The setup of the virulence assay included a 50×50×5 cm arena with a sterile filter paper moistened with 5 mL of sterile distilled water. 10 *P. ibera* slugs were placed into the arena, along with 20 g of fresh lettuce leaves. The arenas were then covered and kept inside of a growth chamber at 18°C, 70% RH, with a 12-hr night and day cycle. The doses of nematodes were pipetted throughout the arena in a suspension of 5 mL. The nematode doses included 250, 500, 1,000, 2,000, and 4,000 IJs/m^2^ (625, 1,250, 2,500, 5,000, or 10,000 IJs per snail, respectively) and molluscicide doses of 1, 2, 4, 8, and 16 g/m^2^, with a negative control of 5 mL of distilled water. The feeding and mortality rate of the slugs were measured daily for 9 days. It is not clear how the authors measured the feeding rate of the slugs. However, it is insinuated that they measured the feeding rate by weighing the slugs each day. All doses of EPNs and molluscicide treatments caused significant mortality compared to the control by day 9. Treatment with molluscicide or *P. hermaphrodita* showed the highest mortality rate with 100% of the slugs killed by day 7 in all doses. However, treatment with *S. carpocapsae* also showed significant mortality with 100% mortality at the 3 highest doses by day 9. *S. feltiae* also had 100% mortality by day 9 at the 2 highest doses. *H. bacteriophora* showed the lowest virulence with about 75% mortality at the highest dose by day 9. The feeding rates of the slugs followed similar patterns seen in the virulence assay. The feeding rate of the slugs was lowest at 0% with the treatment of metaldehyde bait. *P. hermaphrodita* treatment had the next lowest feeding rate, followed by *S. carpocapsae*, *S. feltiae*, and *H. bacteriophora*.

In order to evaluate the damage or grazing incidence, the establishment of seedlings was measured. In total, 55 germinated bean seeds were sown into a 50×50×10 cm container and filled with soil. The same doses of nematodes and molluscicide were applied, and 5 slugs were added to each container. A control was established in this experiment with no slugs or treatments added to the container. The containers were kept on a 12-hr night day cycle at 27°C, 70% RH. The established seedling rate was then determined every other day for 12 days after cultivation. This assay was also repeated twice with five replicates within each trial. The results showed that all EPNs caused a significant increase in the rate of establishment of the seedlings. However, the level of establishment was dependent on the dosage and species of nematodes applied. A similar pattern of species effectiveness in the virulence assay was observed in the seedling establishment assay. The metaldehyde treatment allowed for the most establishment, followed by *P. hermaphrodita*, *S. carpocapsae*, *S. glaseri*, and *H. bacteriophora*. *S. carpocapsae* was only able to increase the establishment of seedlings by about 40% at the highest dose compared to the control. However, at lower, more economical doses of 250 or 500 IJs/cm^2^, seedling establishment was only increased by approximately 20%. EPNs were far less effective than *P. hermaphrodita* (increased by 50-60%) or metaldehyde bait (increased by 80%).

The study by Saeedizadeh and Niasti (2020) provided reasonable evidence that EPNs may be effective biological control agents. They used quantifiable doses over a large range, a reasonably timed assay, and measured aspects of the host biology. However, whether the EPNs parasitized the slugs was not determined. Saeedizadeh and Niasti (2020) did not stage the nematodes after infection. Koch’s postulates were not met, and no aspect of nematode biology was measured. It is not known if the IJs which emerged from the slug cadavers were adults or IJs. The observation of adults or a mixed population of adults and IJs from the slug cadavers would provide evidence that the EPNs parasitized the slug. Regardless, this study demonstrates that *S. carpocapsae* may be an effective biological control agent against *P. ibera*. If 100% mortality of slugs is desired by consumers, a dose of at least 1,000 *S. carpocapsae* IJs/m^2^ would need to be used according to these data. However, a dose of 250 *S. carpocapsae* IJs was shown to cause a mortality rate of about 75%. This is a reasonable mortality rate and may be useful to growers. While this species and dosage is less effective than treatment with *P. hermaphrodita* (Nemaslug®), it is less costly, and therefore may be a more economical option in the future.

## Discussion

EPNs have been well-established as effective biocontrol agents against multiple species of pestiferous insects, many of which have been tested many times by multiple different labs (Kaya and Gaugler, 1993). However, EPNs have not been well-established as biological control agents against gastropods. There are relatively few studies that have been performed with EPNs as a form of control for gastropods. In total, 736 hits appear when the words ‘entomopathogenic nematodes gastropods’ are typed into Google Scholar. Contrastingly, 29,300 hits appear when the words ‘entomopathogenic nematodes insects’ are searched in Google Scholar. This demonstrates the sheer amount of research that has been done on EPNs in relation to insects, and how little research has been done on EPNs in relation to gastropods. Most biological control agents are researched extensively before being commercially produced. It has been established that EPNs are pathogenic to a wide variety of insects across many orders, including: *Diptera*, *Coleoptera*, *Blattodea*, *Hymenoptera*, *Lepidoptera*, *Orthoptera, Siphonaptera*, and *Isoptera* via multiple repeated experiments (Abate et al., 2017; Georgis et al., 2006; Peters, 1996; Shapiro-Ilan et al., 2007). The targeted insects include those from foliar, soil surface, cryptic, and subterranean habitats (Abate et al., 2017; Lacey and Georgis, 2012). EPNs have also been found to be effective against multiple life stages of targeted insects (Abate et al., 2017; Grewal, 2002). In order to determine the efficacy of EPNs versus *Popillia japonica*, *Otiorhynchus sulcatus*, and *Cyclocephala borealis* alone, over 500 field and greenhouse studies were performed (Gaugler and Georgis, 1991).

It is possible that EPNs may function as useful biocontrol agents against gastropods in the future. If they are effective, it remains debatable whether they are a more economically sound choice when compared to Nemaslug® (*P. hermaphrodita*), which is more expensive than EPNs. 5,000,000 EPNs are able to be purchased from Arbico Organics for $61.00, while 3,000,000 *P. hermaphrodita (*Nemaslug) are able to be bought for $40.23. Although the price of EPNs may be more economical, the critical issue is the reduction of snail/slug numbers and prevention of marketable yield losses, not merely the number of IJs released. Another important consideration is the question of non-target effects. Further research is required to determine the efficacy of various EPNs to a variety of gastropod species. We find this area of research to be understudied. Large-scale field trials are nonexistent, limiting any useful conclusions for large-scale growers.

The potential of EPNs in biological control has not been fully realized, and innovations in production, storage, and application could increase their utility. The question of whether EPNs can control gastropod pests and/or vectors of disease has not been adequately explored, though it could lead to increased crop production and decreased human suffering. Optimism has been expressed in this review, based on available published data. However, discussions with colleagues and comments from reviewers suggest that there are unpublished data regarding EPN-gastropod interactions, some of which may be considered negative data. Negative data often goes unpublished in many fields of scientific inquiry, yet it is extremely valuable in revealing what has been attempted and what does not work (Fanelli, 2012; Teixeira da Silva, 2015). These data inform future experiments and allow subsequent research to focus on a narrower set of variables or test alternative hypotheses. Only work that has been published can be reviewed, thus negative data should be published to help inform assessments of biological control agents and strategies moving forward.

**Table 1.** Summarizes which EPN species have been tested on a variety of gastropod species.

Gastropod	EPN species tested	Cited literature
*Bradybaena similaris*	*Heterorhabditis indica*	Tunholi et al. (2014, 2017b)
*Deroceras agreste*	*Heterorhabditis bacteriophora* *Steinernema carpocapsae* *Steinernema feltiae*	Jaworska (1993)
*Deroceras reticulatum*	*Heterorhabditis bacteriophora* *Heterorhabditis sp.* *Steinernema carpocapsae* *Steinernema feltiae*	Jaworska (1993) and Wilson et al. (1994)
*Lymnaea columella*	*Heterorhabditis baujardi*	Tunholi et al. (2017a)
*Oncomenia hupensis*	*Heterorhabditis heliothidis* ^*a*^ *Heterorhabditis heliothidis T327* ^*a*^ *Steinernema bibionis* ^*a*^ *Steinernema glaseri* *Steinernema feltiae*	Li et al. (1986)
*Parmacella ibera*	*Heterorhabditis bacteriophora* *Steinernema carpocapsae* *Steinernema feltiae*	Saeedizadeh and Niasti (2020)

**Note:** ^a^Species names that are junior synonyms and not the currently used species name.
